# Immunomodulatory effects of primed amniotic fluid-derived mesenchymal stem/stromal cells with IFN-γ from unexplained recurrent miscarriage sources

**DOI:** 10.1038/s41598-025-01799-1

**Published:** 2026-01-03

**Authors:** Seyed Mehdi Hoseini, Ahmad Reza Bahrami, Seyed Mehdi Kalantar, Mohammad Hasan Sheikhha, Behrouz Aflatoonian, Nasrin Ghasemi, Elham Sadat Hosseini, Maryam M. Matin, Fateme Montazeri

**Affiliations:** 1 Biotechnology Research Center, Yazd Reproductive Sciences Institute, Shahid Sadoughi University of Medical Sciences and Health Services, Yazd, Iran; 2 Department of Biology, Faculty of Science, Ferdowsi University of Mashhad, Mashhad, Iran; 3 Industrial Biotechnology Research Group, Institute of Biotechnology, Ferdowsi University of Mashhad, Mashhad, Iran; 4 Research and Clinical Center for Infertility, Yazd Reproductive Sciences Institute, Shahid Sadoughi University of Medical Sciences and Health Services, Yazd, Iran; 5 Stem Cell Biology Research Center, Yazd Reproductive Sciences Institute, Shahid Sadoughi University of Medical Sciences and Health Services, Yazd, Iran; 6 Abortion Research Center, Yazd Reproductive Sciences Institute, Shahid Sadoughi University of Medical Sciences and Health Services, Yazd, Iran; 7 Novel Diagnostics and Therapeutics Research Group, Institute of Biotechnology, Ferdowsi University of Mashhad, Mashhad, Iran

**Keywords:** Amniotic fluid, Mesenchymal stem/stromal cells, Paracrine factors, Immunomodulatory capacity, Recurrent pregnancy loss, Cell biology, Developmental biology, Genetics, Immunology, Molecular biology, Stem cells, Diseases, Immunological disorders, Reproductive disorders

## Abstract

The immunomodulatory properties of mesenchymal stem/stromal cells (MSCs) are strongly influenced by IFN-γ priming and may vary depending on their tissue of origin. This study investigates whether the immunoregulatory responses of human amniotic fluid-derived MSCs (hAF-MSCs) to IFN-γ are altered when derived from individuals with unexplained recurrent pregnancy loss (RPL). We analyzed 10 well-characterized hAF-MSC clones, equally divided into non-RPL and RPL groups, to assess IFN-γ-induced expression of immunomodulatory genes. Additionally, we evaluated the paracrine effects of MSC-conditioned medium on T regulatory (Treg) cell induction and macrophage polarization. Significant differences were observed between the two groups in the expression of immunomodulatory markers following IFN-γ priming, as well as in their capacity to induce Treg cells and modulate M1/M2 macrophage polarization. These findings support the hypothesis that dysregulated immune pathways contribute to unexplained RPL by revealing impaired IFN-γ responsiveness in hAF-MSCs from fetuses derived from couples with the history of RPL.

**Supplementary Information:**

The online version contains supplementary material available at 10.1038/s41598-025-01799-1.

## Introduction

As defined in the literature, RPL or recurrent miscarriage is defined by three or more consecutive spontaneous abortions before 20 weeks of gestation^1^. Despite advancements in understanding its etiology, approximately 50% of RPL cases remain unexplained or idiopathic, with no identifiable causative factors. Potential contributors to idiopathic miscarriages include ovarian aging, environmental influences, and immunological dysfunctions^2^. Among these, immunological mechanisms at the fetal-maternal interface have emerged as critical areas of investigation, with implications for both understanding idiopathic miscarriages and developing novel therapeutic approaches, particularly for treating autoimmune diseases^3–6^.

Research into the immunological underpinning of RPL has predominantly focused on analyzing autoantibodies, natural killer cells (NKCs) in blood and decidua, cytokines in circulation or secreted by decidual cells, and classical and non-classical HLA polymorphisms, as well as HLA expression on trophoblastic cells^2,5,7–9^. Recent studies, including our own, have extended this focus to explore the interplay between immunological and genomic factors in RPL, examining maternal, paternal, and combined parental genetic contributions^10^. This has given rise to the hypothesis of “unfavorable genomes,” suggesting that specific combinations of parental genetic profiles may increase the risk of fetal abnormalities and recurrent miscarriage. Unfavorable variants of uncertain significance (VUS), which are not definitively linked to the disease^11–13^, may nonetheless contribute to genomic instability in embryonic cells, reducing the likelihood of achieving a healthy pregnancy in RPL cases^10,11^. Furthermore, evidence suggests that the genomes of products of conception (POCs) in RPL families may harbor genetic predispositions to developmental disorders during pregnancy^12^. Though this hypothesis requires further substantiation, early findings point to potential deficiencies in key elements essential for embryonic cell growth and differentiation in RPL cases^13^.

MSCs are increasingly recognized as pivotal regulators of immunological mechanisms, capable of switching between pro-inflammatory (MSC1) and immunomodulatory (MSC2) phenotypes in response to pro-inflammatory cytokines such as IFN-γ, TNF-α, IL-1β, and IL-17^14,15^. Inflammatory microenvironments activate tissue-resident MSCs and recruit circulating MSCs to sites of tissue damage, where they promote regeneration and restore homeostasis^16,17^. The dynamic regulation of immune responses by MSCs is a critical aspect of tissue recovery, mediated through innate immune activation, chemokine upregulation, adaptive immune cell recruitment, and the licensing of MSCs to acquire an immunomodulatory phenotype (MSC2)^17–21^. Disruptions in this intricate process, potentially influenced by genomic variations, may impair immunoregulation and possibly contribute to RPL pathogenesis^22^.

Given this context, we hypothesize that the source of MSCs influences their immunomodulatory capacity in response to IFN-γ. Amniotic fluid contains a heterogeneous population of differentiated, progenitor, and multipotent fetal cells derived from various fetal and extra-embryonic tissues^23–25^. Numerous studies have investigated AF-MSCs for diverse therapeutic and research applications^26,27^. As AF-MSCs are representative of fetal cells that reflect the parental genomic background, an intriguing question arises: could the genomic background of couples with RPL result in fetuses with AF-MSCs that exhibit altered immunomodulatory responses to IFN-γ compared to those from unaffected pregnancies?

## Materials and methods

### Sampling and experimental design

Human amniotic fluid samples were collected by ultrasound through the procedure of amniocentesis at the second trimester, ranging from 16th to 18th weeks of pregnancy. The application of specimens in this study did not interfere with the prenatal diagnosis (PND) process; nonetheless, informed consent was acquired from participants, particularly due to the inclusion of vulnerable groups, i.e. women who have suffered from recurrent miscarriages. It should be noted that all methods were conducted in accordance with the strict biological ethics guidelines of the Yazd Reproductive Sciences Institute.

The clinical characteristics of mothers and their fetuses are presented in the Supplementary Table 1. These samples were included in the study based on the normal karyotype of fetus and maternal age of 27–34 years. Several specimens were excluded from the study due to high-risk pregnancies that could have influenced the results. These included cases involving tobacco and alcohol use, as well as maternal diseases such as autoimmune disorders, preeclampsia, and gestational diabetes.

The study included two groups. The first group, which served as a healthy control, was comprised of five pregnant women who had not experienced any miscarriages and had at least one full-term pregnancy (non-RPL group). The second group consisted of five pregnant women who had a history of idiopathic recurrent miscarriage (RPL group). The AF-MSC samples in both non-RPL and RPL groups were evaluated for their immunoregulatory properties under five different conditions. These included a basic culture without stimulation with pro-inflammatory cytokine IFN-γ as the control. Additionally, the IFN-γ stimulated state was evaluated at two concentrations of 20 and 100 international units per milliliter (IU/ml) of culture medium, in two time-intervals of 24 and 72 h.

### Derivation of mesenchymal stem/stromal cells from human amniotic fluid

With the aim of not interfering with the PND process, we implemented a two-step protocol for obtaining AF-MSCs. Our previous findings demonstrated that this method is the most convenient and effective option with the least impact on PND^28^. To derive AF-MSCs, amniocytes were sequentially cultured in a T25 flasks with a density of 10^4^ cells/cm^2^ at 37 ℃ and under 5% CO_2_. For this purpose, a modified medium composed of 2:1 v/v Dulbecco’s modified Eagle’s medium (DMEM)-AmnioMAX-II was used as a superior medium compared to either DMEM or AmnioMAX-II^25^. The cells were harvested at the third passage for characterization by flow cytometry analysis and mesodermal differentiation capacity, as well as experimental cultures in different stimulatory mimetic situations.

### Cytogenetic analysis

To ensure that the AF-MSCs are chromosomally stable, the clones were screened for any numerical or structural abnormalities. The cells were harvested at P3 using Carnoy fixative solution, within 24 to 48 h after the sub-culturing process, depending on the growth rate. The slides were prepared, followed by banding and staining with Giemsa (Sigma-Aldrich Co., Germany), and then used for standard chromosome G-banding analysis.

### Flow cytometry analysis

Based on the International Society for Cell & Gene Therapy (ISCT) minimal criteria, MSCs are classified as clonal cells expressing mesenchymal markers, including CD44, CD73, CD90, and CD105, while they lack the expression of hematopoietic markers, such as CD31, CD34 and CD45^29^. For this purpose, the expanded clones were characterized by flow cytometry regarding the presence of mesenchymal surface markers, i.e., CD44, CD90, and CD105. The vascular endothelial marker CD31 was also analyzed as a negative indicator. To do so, at least 10^5^ cells for each assay were detached from T25 flasks by trypsin-EDTA treatment and the cell pellet was washed twice in phosphate-buffered saline (PBS) containing 0.2% fetal bovine serum (FBS). Cells were then incubated with the following antibodies in PBS supplemented with 1% bovine serum albumin (BSA), including FITC-conjugated anti-human CD44 and CD90, as well as PE-conjugated anti-human CD105 and CD31 (all from Immunostep).

In order to detect induced Treg cells and macrophage polarization, human peripheral blood mononuclear cells (PBMCs) cultured doubly in MSC-conditioned medium (MSC-CM) were analyzed by flow cytometry regarding the target markers. For this purpose, PBMCs were incubated with Treg-specific conjugates in PBS supplemented with 1% BSA. These markers included PerCP-conjugated anti-human CD4, APC-conjugated anti-human CD25, and PE-conjugated anti-human FOXP3 antibodies. The target markers for macrophage polarization were examined using PE.Cy7-conjugated anti-human CD68, FITC-conjugated anti-human CD80, and PE-conjugated anti-human CD206 antibodies. The conjugates were purchased from Immunostep, BioLegend, and Exbio. The results were analyzed on a BD FACSCalibur and the graphs were generated in FlowJo (v 10.1, Tree Star, Inc.) software.

### Mesodermal differentiation

Differentiation ability of clonal cells into mesodermal lineages was examined by assessing adipogenic and osteogenic differentiation. The expanded cells were detached from T25 flasks in third passage, counted and then seeded in 6-well plates at a density of 5 × 10^3^ cells/cm^2^ in DMEM containing 10% FBS and 1% penicillin-streptomycin (Pen-Strep) as basal medium. At 70% confluency, the cultures were incubated under differentiation media for 3 weeks (refreshing medium every 3 days). For adipogenic induction medium, the basal medium was supplemented with 50 µg/ml indomethacin, 50 µg/ml ascorbic acid-2-phosphate, 10 IU/ml insulin and 100 nM dexamethasone. Further, for osteogenic differentiation, 10 mM β-glycerol phosphate, 50 µg/ml ascorbic acid-2-phosphate and 10 nM dexamethasone were added to the basal medium (all supplements were from Sigma-Aldrich). To confirm adipogenesis in the cells, intracellular lipid vacuoles were stained with oil red O (Sigma-Aldrich). Validation of osteogenesis was performed *via* visualization of extracellular matrix mineralization by alizarin red S^30^.

### Molecular analysis

The quantitative RT-PCR analysis was used to evaluate the response of AF-MSCs to IFN-γ under five priming treatments. For this purpose, AF-MSCs from non-RPL and RPL groups were assessed for the expression levels of immunoregulatory genes, including indoleamine 2,3-dioxygenase 1 and 2 (*IDO1* and *IDO2*), leukemia inhibitory factor (*LIF*), transforming growth factor-beta (*TGF-β*), interleukin-6 and − 10 (*IL-6* and *IL-10*), cyclooxygenase 1 and 2 (*COX1* and *COX2*), Toll-like receptor 4 (*TLR-4*), and vascular cell adhesion molecule 1 (*VCAM1*)^24,31^. *18 S rRNA* was used as a reference gene in all RT-PCR reactions. All human-specific primers were designed for intron-spanning regions. Primer sequences, their melting temperatures (Tm) and the size of amplified products are listed in Table 1.

**Table 1:** **)** characteristics of the primers designed to check the expression of target genes in this study.

Gene	Primer sequence	Tm (°C)	Product size (bp)
*IDO1*	F: GGCAAAGGTCATGGAGATGT R: TCCAGTTTGCCAAGACACAG	58 58	127
*IDO2*	F: CTGGTCCTGAGCTTCCTCAC R: CAGCACCAAGTCTGAGTGGA	59 59	153
*LIF*	F: ACCAGATCAGGAGCCAACTG R: GCCACATAGCTTGTCCAGGT	60 60	113
*IL-6*	F: AGTGAGGAACAAGCCAGAGC R: GTTGGGTCAGGGGTGGTTAT	59 59	104
*TGF-β*	F: TTGATGTCACCGGAGTTGTG R: GAACCCGTTGATGTCCACTT	60 59	125
*IL-10*	F: CCAAGACCCAGACATCAAGG R: AAGGCATTCTTCACCTGCTC	59 59	138
*COX1*	F: GAGCAGCTTTTCCAGACGAC R: GCAGGAAATAGCCACTCAGC	59 60	97
*COX2*	F: CCCATGTCAAAACCGAGGTG R: TCCGGTGTTGAGCAGTTTTC	59 59	102
*VCAM-1*	F: ATGGAATTCGAACCCAAACA R: CCTGGCTCAAGCATGTCATA	60 59	139
*TLR4*	F: ACCTCCCCTTCTCAACCAAG R: TGTCTGGATTTCACACCTGGA	59 59	125
*18 S rRNA*	F: AGAAACGGCTACCACATCCA R: CCCTCCAATGGATCCTCGTT	59 59	158

Following qualitative assessment of total RNA extracted from AF-MSCs by TRIzol reagent (Roche), 100 ng of total RNA was used to synthesize cDNA using the RevertAid First Strand cDNA Synthesis Kit (Thermo Fisher Scientific). Applied Biosystems^®^ SYBR^®^ Green PCR Master Mix was used for preparation of RT-PCR reactions. Quantitative analysis was carried out by means of Applied Biosystems StepOnePlus Real-Time PCR System. To do this, real-time system was set on initial denaturation step at 95 ℃ for 10 min, followed by 40 cycles of 95 ℃ for 20 s, 58–60 ℃ (based on different annealing temperatures of primers) for 20 s and 72 ℃ for 30 s.

### Induction of Treg cells by MSC-conditioned medium

To evaluate the effects of MSC-CM on Treg cell induction, peripheral blood (PB) was collected from two healthy male donors aged 23 and 25 years. Donors were selected based on their general health status, including the absence of severe pathological conditions such as autoimmune diseases, and were screened for current infections using routine blood tests. These tests included a complete blood count (CBC) and additional indicators such as red and white blood cell counts, platelet counts, hemoglobin, hematocrit, lipid panel (LDL, HDL), metabolic panel (calcium, glucose, sodium, potassium), thyroid panel (T3, T4, TSH), and C-reactive protein (CRP) levels. The donors were included in the study due to their normal blood test results.

PBMCs were isolated from 15 ml of PB using a density gradient method with Lymphocyte Separation Solution (Serana), following the manufacturer’s protocol. The isolated PBMCs were cultured at a concentration of 10^6^ cells/ml in Roswell Park Memorial Institute (RPMI) 1640 medium supplemented with 10% FBS, 1% Pen-Strep, and 1 µg/ml phytohemagglutinin (PHA; Gibco) for 48 h to stimulate mitogenic activation. After culture, PHA-stimulated PBMCs were harvested by centrifugation, washed with 5 ml PBS, and centrifuged again. The resulting cell pellet was resuspended and cultured in MSC-CM (10^6^ cells/ml) for 72 h to assess Treg cell induction.

For MSC-CM preparation, the culture media from AF-MSC clones were collected separately and stored at − 80 °C. MSC-CM from clones of non-RPL and RPL groups were pooled into five distinct treatment conditions. To serve as a control for Treg induction efficiency, PHA-stimulated PBMCs were cultured in a modified medium comprising DMEM-AmnioMAX-II (2:1 v/v) supplemented with recombinant human TGF-β1 (R&D Systems) at a final concentration of 10 ng/ml, as previously described^32^.

### Induction of M2 macrophages by MSC-conditioned medium

PHA-stimulated PBMCs from the same healthy donors were used to evaluate the effects of MSC-CM on macrophage polarization. The PHA-stimulated PBMCs (10^6^ cells/ml) were cultured in MSC-CM for 72 h to assess its impact on macrophage phenotype. To serve as a control for inducing M2 macrophages, PHA-stimulated PBMCs were cultured in a modified medium consisting of DMEM-AmnioMAX-II (2:1 v/v), supplemented with recombinant human TGF-β1 at a final concentration of 10 ng/ml, based on previously reported protocols^33^.

The impact of MSC-CM on both Treg induction and macrophage polarization was analyzed using flow cytometry, as detailed in section “2.4. Flow cytometry analysis”.

### Statistical analysis

The RT-PCR results were quantitatively assessed using the 2^−∆∆CT^ method. This method involves analyzing the difference between the reference and target C_T_ values of each sample. The expression levels of five mimetic states were analyzed using two-way analysis of variance (ANOVA) to evaluate all treatments together. Additionally, the non-parametric Mann-Whitney test was used for dual analysis of treatments. Pearson’s coefficients were calculated to identify possible differences in the correlation of target genes between the non-RPL and RPL groups. In all statistical analyses, significant level was set at *p*-value < 0.05.

## Results

### Characterization of mesenchymal stem/stromal cells derived from amniotic fluid

Adherent cells derived from amniotic fluid were isolated, expanded to the third passage, and analyzed to confirm their MSC characteristics and chromosomal stability. Flow cytometry revealed that more than 90% of the cells in both the non-RPL (healthy control) and RPL (case) groups expressed the MSC markers CD44, CD90, and CD105. Additionally, over 97% of the cells were negative for CD31, indicating the absence of endothelial or hematopoietic contamination. The flow cytometry results for non-RPL and RPL clones are detailed in Supplementary Figs. 1 and 2, respectively.

To confirm the differentiation capacity of the derived MSCs, all clones were cultured under adipogenic and osteogenic conditions. Cells from both groups successfully differentiated into adipocytes and osteocytes, as shown in Supplementary Fig. 3 (Panels A and B). Cytogenetic evaluations further confirmed the normal karyotype of all 10 clones included in the study, with representative karyograms provided in Supplementary Fig. 4. Two clones, one from each group, were excluded from the study due to trisomy 21 chromosomal abnormalities observed during the initial screening.

### **Expression of immunoregulatory genes in response to IFN-γ**

**Fig. 1:**
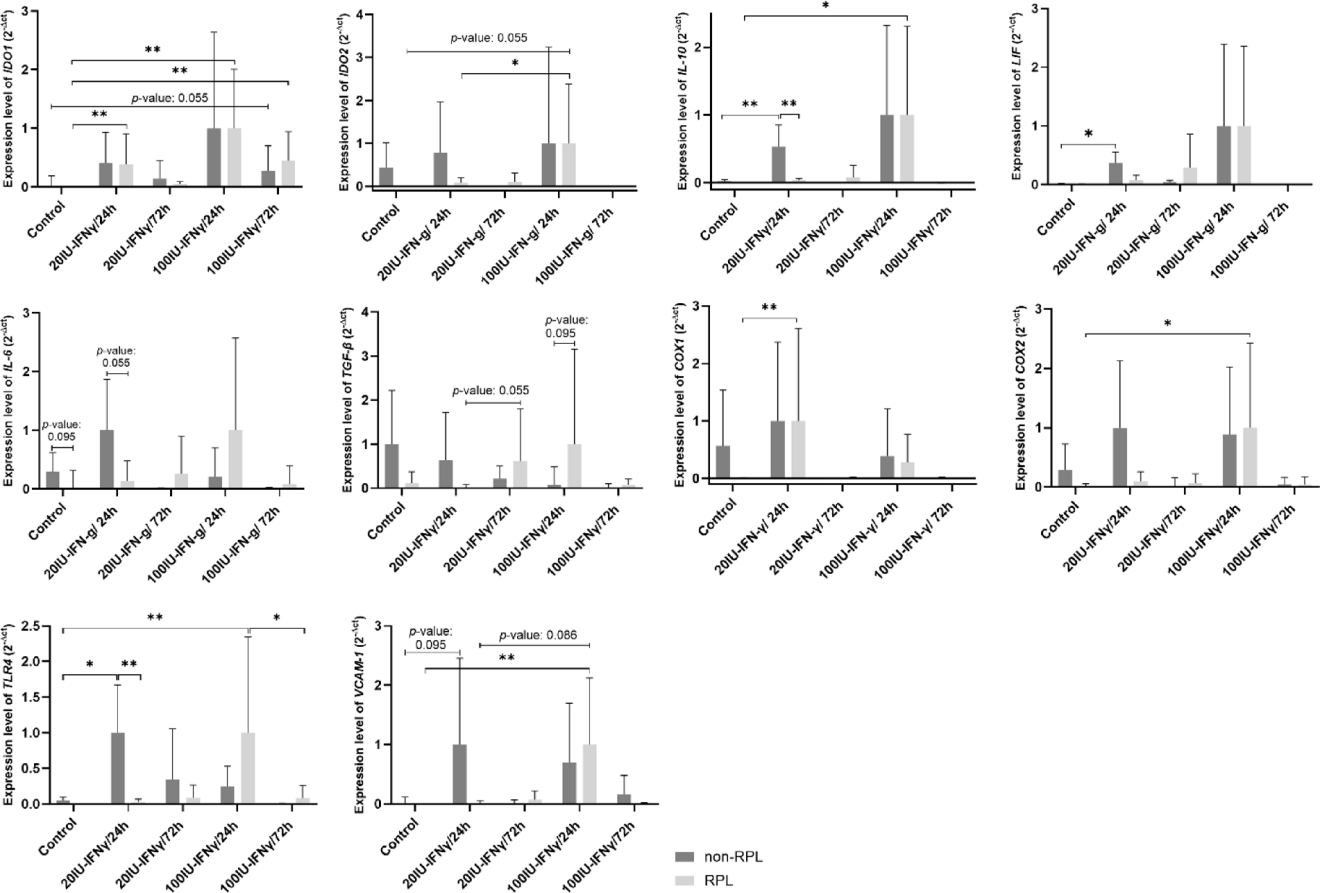
Expression of target genes in response to IFN-γ. The expression level of immunomodulatory genes, *IDO1*, *IDO2*, *IL-10*, *LIF*, *IL-6*, *TGF-β*, *COX1*, *COX2*, *TLR4*, and *VCAM-1* in AF-MSCs derived from samples of non-RPL and RPL groups under five different treatments; control (non-preconditioned by IFN-γ), preconditioned with two different concentrations of IFN-γ (20 IU/ml and 100 IU/ml) after two time-intervals (24 h and 72 h). The averages of five clones were analyzed using the Mann-Whitney test as dual comparisons. The *p*-values of non-significant differences (close to the significant level, i.e., < 0.1) are written above the compared column bars. The significant levels are marked as *p*-values < 0.05 (*) and < 0.01 (**).

The study assessed the relative expression of immunomodulatory genes (*IDO1*, *IDO2*, *IL-10*, and *LIF*) under various inflammatory-mimetic conditions (Fig. 1). ANOVA analysis revealed significant differences among treatments for *IDO1* (*p* = 0.023), *IL-10* (*p* = 0.002), and *LIF* (*p* = 0.006), while differences for *IDO2* were not statistically significant (*p* = 0.12).

In the RPL group, *IDO1* expression significantly increased following treatments with 20 IU/ml-24 h and 100 IU/ml (both 24 h and 72 h) IFN-γ, compared to the control group (*p* < 0.01). However, these treatments had no significant impact on *IDO1* expression in the non-RPL group. A similar trend was observed for *IDO2*, *IL-10*, and *LIF*: non-RPL clones responded moderately to 20 IU/mL-24 h IFN-γ treatment, whereas RPL clones showed significant upregulation with increasing IFN-γ doses or prolonged exposure (*p* < 0.05).

Expression of pleiotropic cytokines *IL-6* and *TGF-β* also displayed distinct patterns between the groups. Non-RPL clones exhibited higher baseline expression levels and moderate responses to 20 IU/mL-24 h IFN-γ treatment, followed by reductions at higher doses and prolonged exposures. In contrast, RPL clones displayed minimal baseline expression but showed substantial upregulation with higher IFN-γ doses (100 IU/mL-24 h) or extended treatment times (72 h).

As illustrated in Fig. 1, the relative expression of *COX2*, *TLR4*, and *VCAM-1* in response to IFN-γ exhibited a consistent trend, showing a significant increase from the control to the 20 IU/mL-24 h treatment in the non-RPL group. Similarly, in the RPL group, the expression levels were significantly upregulated in the 100 IU/mL-24 h treatment compared to the controls (*p* < 0.05). However, *COX1* displayed a distinct behavior compared to these three genes—*COX2*, *TLR4*, and *VCAM-1*. Specifically, the response to IFN-γ was similar in both non-RPL and RPL groups. Despite this similarity, the high baseline expression of *COX1* in the controls of the non-RPL group resulted in a non-significant increase in expression after the 20 IU/mL-24 h treatment. Conversely, the absence of *COX1* expression in the controls of the RPL group led to a significant upregulation from the control to the 20 IU/mL-24 h treatment (*p* < 0.05).

### Overview of expression profiles and correlations among genes

**Fig. 2:**
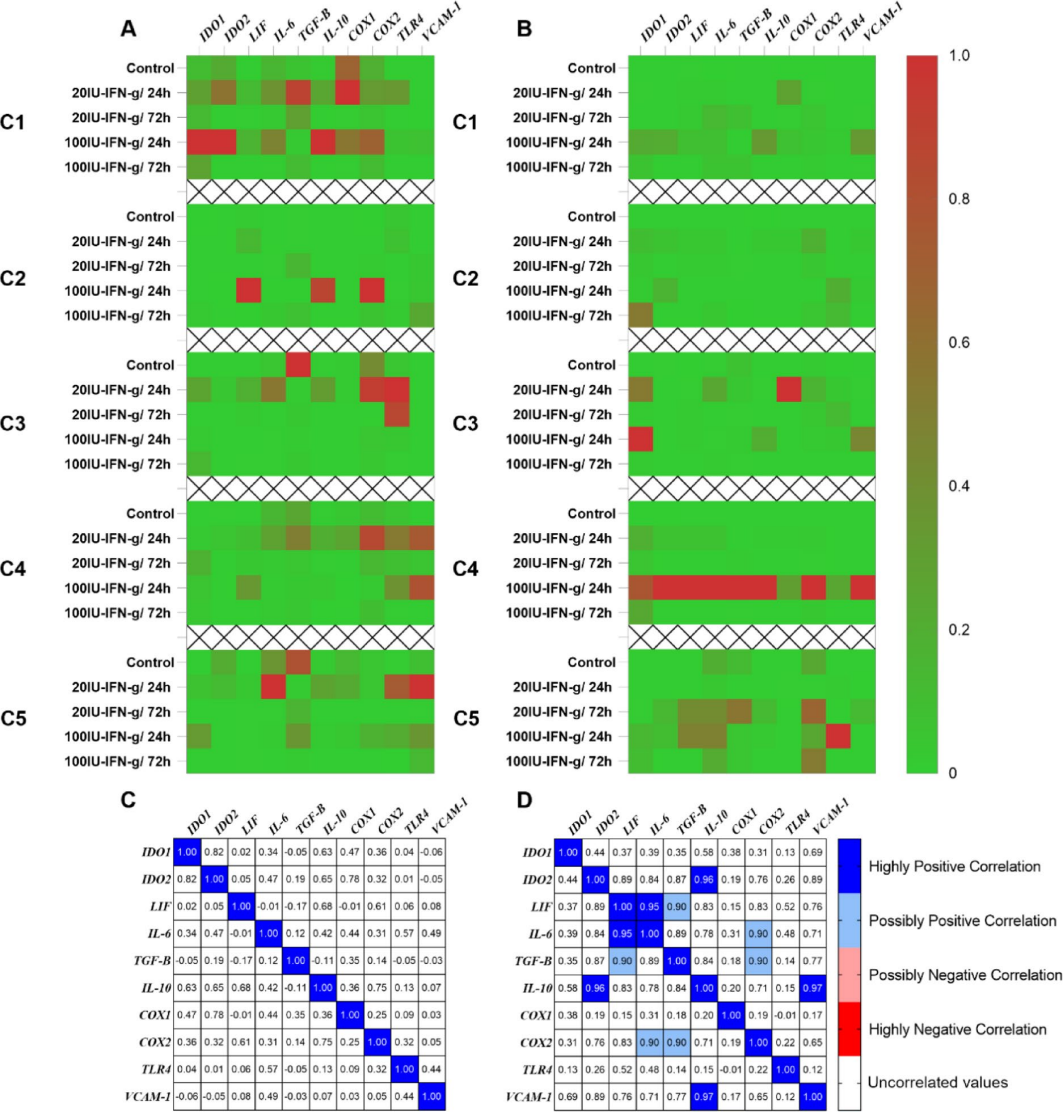
Overview of expression profiles and correlations among target genes. The heatmaps represent a descriptive overview of the expression level of target genes across different treatments in both non-RPL (**A**) and RPL (**B**) groups, in which the data have been normalized so that the lowest and highest values are represented as 0 and 1, respectively. To show any possible relationships between the levels of target genes and their responses to different treatments, in non-RPL (**C**) and RPL (**D**) groups, Pearson correlation analysis was used based on the Pearson coefficient within the significant range (*p*-values < 0.05). Correlations were classified based on the Pearson coefficient, with highly positive ranging from 0.95 to 1, possibly positive ranging from 0.90 to < 0.95, possibly negative ranging from >-0.95 to -0.90 and highly negative ranging from − 1 to -0.95. Non-significant correlations (Pearson coefficient: >-0.90 and < 0.90) are excluded in the plots as white cells.

Heatmaps (Fig. 2) were generated to visualize the relative expression of target genes across individual clones (C1–C5) and treatments in both non-RPL (Fig. 2A) and RPL (Fig. 2B) groups. Positive correlations were represented by Pearson’s coefficient (*r*) of “0.9 to 1”, while values of “-0.9 to -1” were indicated as negative correlations, provided they were within the significant range (*p*-values < 0.05). Data normalization ensured that the lowest and highest values were represented as 0 and 1, respectively. The heatmaps revealed clear differences between groups: non-RPL clones exhibited moderate responses to varying IFN-γ doses and exposure times (Fig. 2A; C1-C5), while RPL clones showed more pronounced and dose-specific induction of target genes (Fig. 2B; C1-C5). For instance, non-RPL clones maintained *COX1* and *COX2* expression under control conditions, whereas RPL clones exhibited minimal baseline expression, with strong induction observed only after specific IFN-γ treatments.

Pearson correlation analyses further highlighted group-specific relationships among gene expression profiles (Fig. 2C and D). In the RPL group, strong positive correlations were observed between *IL-10* and *IDO2* (*r* = 0.96), as well as between *IL-10* and *VCAM-1* (*r* = 0.97). Similarly, *LIF* correlated positively with *IL-6* (*r* = 0.95), while moderate correlations were noted between *TGF-β* and both *LIF* and *COX2* (*r* = 0.90–0.95). In contrast, the non-RPL group showed no significant correlations among the genes.

These findings suggest a distinct, dysregulated response to IFN-γ in RPL clones, potentially driven by genomic or immunological abnormalities. The observed correlations in the RPL group may reflect underlying mechanisms contributing to altered immunoregulation in these cases.

### Comparison of the Immunomodulatory capacity in paracrine mode

#### Treg cell induction in PBMCs under MSC-conditioned medium

**Fig. 3:**
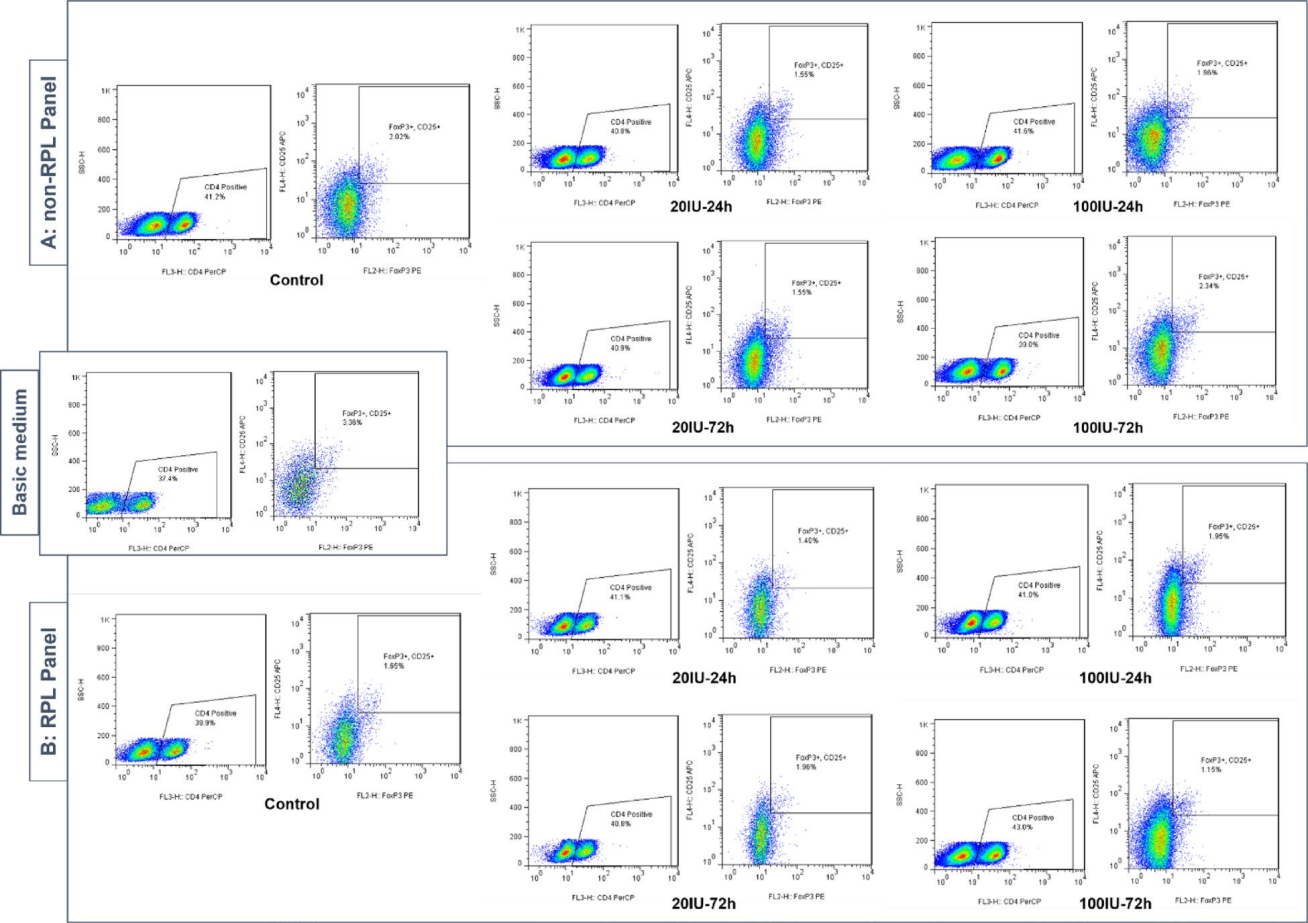
The density dot plots from flow cytometry analysis representing Treg gates. The results are depicted in 3 boxes: (**A**) non-RPL and (**B**) RPL Panels (five upper and lower double-dot plots, respectively) for induction of Treg cells by MSC-CM in different treatments, including control (CM from unstimulated MSCs), 20 and 100 IU/ml of IFN-γ during 24 h and 72 h. One double-dot plot depicted in a single box in the middle for induction of Treg cells by the basic medium (TGF-β1-supplemented RPMI 1640). PBMCs were gated based on lymphocyte size and granularity using forward scatter (FSC) and side scatter (SSC) parameters (not presented here). Next, CD4⁺ lymphocytes were identified within this gated population, represented in the trapezoid region of the left dot plot in each binary plot. Finally, CD25⁺FOXP3⁺ cells were selected from the CD4⁺ population, represented in the square region of the right dot plot in each binary plot.

PBMCs were isolated from two healthy young donor to investigate the induction of regulatory T cells (Treg; CD4⁺CD25⁺FOXP3⁺) under the influence of MSC-CM. Figure 3 displays the Treg induction across different conditions. The Treg induction by the basic medium (RPMI 1640 supplemented with 10 ng/ml TGF-β1) and pooled MSC-CMs from five clones in the non-RPL (Fig. 3-Panel A) and RPL (Fig. 3-Panel B) groups is illustrated. The percentage of CD4⁺CD25⁺FOXP3⁺ Treg cells was determined using a three-step gating strategy. First, PBMCs were gated based on lymphocyte size and granularity using forward scatter (FSC) and side scatter (SSC) parameters. Next, CD4⁺ lymphocytes were identified within this gated population, represented in the trapezoid region of the left dot plot in each binary plot. Finally, CD25⁺FOXP3⁺ cells were selected from the CD4⁺ population, represented in the square region of the right dot plot in each binary plot (Fig. 3).

Figure 5 further quantifies Treg induction, presenting the percentages of Treg cells (Fig. 5A) and Treg/CD4⁺ T cell ratios (Fig. 5B) as bar graphs. The percentage of Treg cells was highest in the basic medium and lower in both MSC-CM groups. While Treg induction levels were comparable between non-RPL and RPL groups under most conditions (24 h treatments with both IFN-γ concentrations and 72 h treatment with 20 IU/ml IFN-γ), a significant reduction in Treg induction was observed in the MSC-CM derived from the control (*p* = 0.012) and 100 IU/mL-72 h (*p* = 0.011) treatments of the RPL group compared to the non-RPL group (Fig. 5A). Similarly, the Treg/CD4⁺ T cell ratios followed the same trend. Ratios were significantly lower in the MSC-CM derived from the control (*p* = 0.012) and 100 IU/mL-72 h (*p* = 0.004) treatments of the RPL group compared to the non-RPL group (Fig. 5B).

#### Macrophage polarization in PBMCs under MSC-conditioned medium

**Fig. 4:**
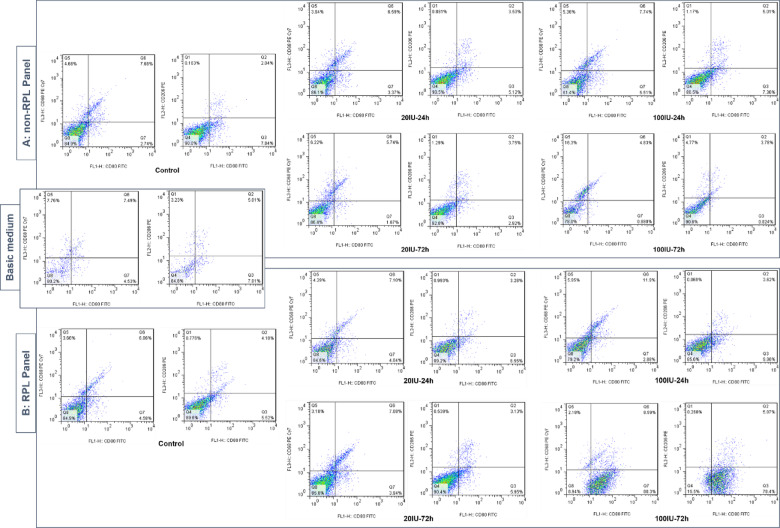
The density dot plots from flow cytometry analysis representing macrophage gates. The results are depicted in 3 boxes: (**A**) non-RPL and (**B**) RPL Panels (5 upper and lower double-dot plot, respectively) for polarization of macrophages by MSC-CM in different treatments, including control (CM from unstimulated MSCs), 20 and 100 IU/ml of IFN-γ during 24 h and 72 h. One double-dot plot depicted in a single box in the middle represents the polarization of macrophages by the basic medium (TGF-β1-supplemented RPMI 1640). The results of each double-dot plot depict the gating of main population based on the markers CD68 and CD80 on the left and CD80 and CD206 on the right. The main population is gated based on FSC and SSC parameters, which their plots are not presented here. FSC: forward scatter; SSC: side scatter.

**Fig. 5:**
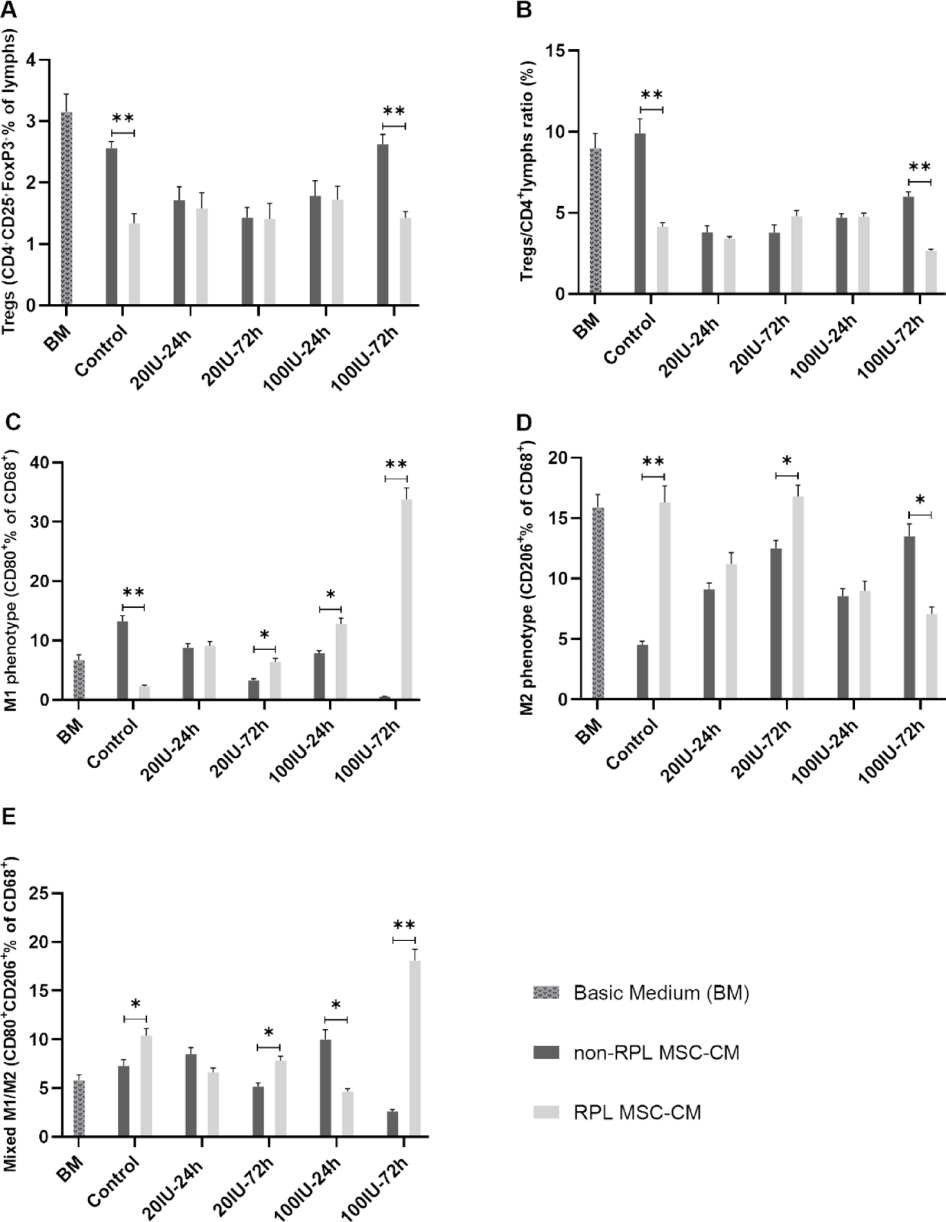
The bar graphs representing the results of flow cytometry analysis. The results of Treg cell induction and macrophage polarization by MSC-CM are presented in different treatments, including basic medium (BM) TGF-β1-supplemented RPMI 1640, control (CM from unstimulated MSCs), 20 and 100 IU/ml of IFN-γ during 24 h and 72 h. (**A**) The proportion of Treg cells as the fraction of CD25⁺FOXP3⁺ cells in the CD4⁺ cell population, as described in the legend of Fig. 3; (**B**) The ratio of CD25⁺FOXP3⁺ Treg cells to CD4⁺ cells; (**C**) The proportion of M1 macrophages in the main population based on the fraction of CD68⁺CD80⁺ cells; (**D**) The proportion of M2 macrophages in the main population based on the fraction of CD68⁺CD206⁺ cells; (**E**) The proportion of mixed M1/M2 macrophages in the main population based on the fraction of CD68⁺CD80⁺CD206⁺ cells.

The study aimed to evaluate the effect of MSC-CM on macrophage polarization. To determine the proportions of M1 and M2 macrophage phenotypes, CD68, CD80, and CD206 markers were analyzed. The polarization data, derived from pooled MSC-CM related to five clones, are presented in dot plots in Fig. 4, with separate panels for non-RPL (Fig. 4A) and RPL (Fig. 4B) groups. For comparison, macrophage polarization in the basic medium (TGF-β1-supplemented RPMI 1640) was used as a control. The summarized results from these markers are depicted as bar graphs in Fig. 5 (C-E) for clarity. The analysis focused on CD68⁺ cells (total macrophages/monocytes) to assess the proportions of CD80⁺ (M1 macrophages) and CD206⁺ (M2 macrophages) populations.

The MSC-CM derived from the control treatment significantly increased the proportion of CD68⁺CD80⁺ (M1) cells in the non-RPL group compared to the RPL group (*p* = 0.004, Fig. 5C). Conversely, the treatments with 20 IU/ml for 72 h (*p* = 0.020) and 100 IU/ml for both 24 h (*p* = 0.021) and 72 h (*p* = 0.001) showed significantly higher M1 polarization in the RPL group compared to the non-RPL group. For M2 polarization, the proportion of CD68⁺CD206⁺ cells significantly increased in the control (*p* = 0.007) and the 20 IU/ml 72 h (*p* = 0.033) treatments of the RPL group compared to the non-RPL group (Fig. 5D). However, in the 100 IU/ml 72 h treatment, M2 polarization was significantly higher in the non-RPL group compared to the RPL group (*p* = 0.016). To investigate the mixed M1/M2 phenotype, the co-expression of CD80 and CD206 was analyzed. The proportion of CD80⁺CD206⁺ cells was significantly higher in the control (*p* = 0.045) and in the 72 h treatments of both 20 IU/ml (*p* = 0.024) and 100 IU/ml (*p* = 0.002) in the RPL group compared to the non-RPL group (Fig. 5E). However, the 100 IU/ml 24 h treatment showed a significantly higher mixed phenotype in the non-RPL group compared to the RPL group (*p* = 0.016).

## Discussion

Unexplained or idiopathic miscarriages are largely attributed to defective mechanisms in the acceptance of the semi-allograft fetus by the maternal uterine environment, potentially associated with immunological processes^2,34^. While studies have focused on maternal immune dysfunction and tolerogenic mechanisms, the potential role of fetal factors in RPL remains underexplored. Emerging evidence suggests that RPL may stem from genetic contributions, particularly those associated with immunoregulatory processes^10^. The “unfavorable genomes” hypothesis proposes that genetic instability resulting from maternal and paternal genome interactions may impair embryonic viability and compromise immunoregulatory functions of fetal cells^10–12^. As a result, modifying the immunoregulatory functions of fetal cells may be a potential outcome of unfavorable genetic compositions.

Developmental abnormalities have been consistently observed in aborted fetuses from RPL cases, even in the absence of chromosomal anomalies, indicating severe growth and morphogenesis disturbances^35^. Furthermore, studies have linked maternal RPL history to increased risks of birth defects (4.2% vs. 2.5%)^36^, as well as long-term neurological, developmental, and movement disorders in offspring^37^. Similar patterns have been reported in other conditions, such as respiratory morbidity, suggesting a shared immunological etiology^38^. These findings inspired our hypothesis that cellular-level abnormalities, particularly in fetal stem cells, may also be present in RPL cases. Accordingly, we focused on AF-MSCs as representative models of fetal MSCs to explore potential differences between RPL and non-RPL groups.

MSCs exhibit remarkable plasticity, dynamically adapting their immunoregulatory functions in response to environmental cues. These cues polarize MSCs into either a pro-inflammatory (MSC1) or anti-inflammatory (MSC2) phenotype^39^. This adaptability allows MSCs to function as “sensors and switchers,” amplifying inflammatory responses in hypoactive immune states or mitigating excessive inflammation to restore immune homeostasis in hyperactive systems^40^. The polarization process is orchestrated by a sophisticated network of cytokines, chemokines, and cell-surface receptors, enabling MSCs to effectively modulate immune responses and contribute to tissue repair^14^. A recent comprehensive review by Tan et al.. underscores the impact of IFN-γ dosing on MSC polarization^41^. The study highlights that priming MSCs with IFN-γ at concentrations between 10 and 100 ng/mL for 24 to 48 h markedly enhances their immunosuppressive capabilities. Specifically, this priming induces the expression of IDO through the Janus kinase (JAK)-signal transducer and activator of transcription 1 (STAT1) pathway. As a result, MSCs exhibit increased suppression of T-cell proliferation and a shift toward the anti-inflammatory MSC2 phenotype. However, insufficient IFN-γ concentrations fail to elicit this immunosuppressive state, while excessive levels may compromise MSC viability and functionality. This dose-dependent response emphasizes the critical role of cytokine signaling in modulating MSC therapeutic potential.

In inflammatory contexts, interactions between tissue-resident cells, immune cells, and MSCs play a critical role in determining the inflammatory state. Dysregulated cytokine levels can skew MSC polarization, contributing to autoimmune diseases^42^. For instance, type 1 diabetes mellitus (T1DM) patients and their relatives exhibit heightened baseline inflammation, leading to amplified responses to inflammatory stimuli^43^. Our previous work demonstrated that AF-MSCs co-cultured with PBMCs from T1DM patients showed altered chemokine expression and Treg induction compared to PBMCs from healthy donors. RPL patients may also experience a similar dysregulated inflammatory microenvironment that impairs the immunomodulatory capacity of MSCs, which play a key role in regulating immune responses at inflamed sites. Our study supports this notion by analyzing gene expression levels under different treatments, revealing distinct patterns between RPL and non-RPL clones.

Specifically, most target genes in RPL clones showed a pronounced response to only one treatment, while remaining unresponsive to others. In contrast, non-RPL clones exhibited a more consistent, mild expression of these genes across various treatments. A comparison of target gene expression between non-RPL and RPL clones demonstrated significant differences, likely stemming from the varied responses of AF-MSCs to different concentrations and exposure durations of IFN-γ. Notably, RPL clones required higher doses of IFN-γ to induce expression of most target genes, resulting in markedly elevated expression levels compared to non-RPL clones. This included genes such as *IDO2*, *IL-10*, *LIF*, *IL-6*, *TGF-β*, *COX2*, *TLR4* and *VCAM-1*, as illustrated in Fig. 1. Moreover, the duration of IFN-γ exposure significantly influenced gene expression patterns between the two groups. In the non-RPL group, genes such as *IDO2*, *LIF*, *IL-6*, *TGF-β*, *IL-10*, *COX1*, and *COX2* were downregulated when exposure time increased from 24 to 72 h at a dosage of 20 IU/mL. In contrast, these genes in the RPL group showed either stable or increased expression levels under the same conditions. These findings highlight the altered responsiveness of AF-MSCs in RPL patients, suggesting a disrupted capacity to adapt to inflammatory cues, which may contribute to their impaired immunoregulatory function.

The heatmap and correlation analysis revealed notable differences in gene expression and regulatory patterns between non-RPL and RPL clones, highlighting a distinct response to IFN-γ in the RPL group (Fig. 2A and B). Non-RPL clones displayed moderate and consistent responses across varying doses and exposure times, leading to variable levels of target genes among treatments. In contrast, RPL clones exhibited minimal baseline expression of key genes, such as *COX1* and *COX2*, with sharp induction under specific IFN-γ treatments. These variable responses to IFN-γ observed in RPL clones resulted in modified correlations of some target genes, as evidenced by Pearson’s coefficient analysis (Fig. 2C and D). Strong positive correlations, particularly between *IL-10* and *IDO2*, and *IL-10* and *VCAM-1*, may imply a dysregulated network of immunomodulatory genes in RPL clones. This altered gene expression profile may reflect genomic or immunological abnormalities that disrupt the normal interplay of cytokines and regulatory molecules, contributing to the immune niches associated with RPL. While these findings suggest possibly impaired sensitivity of RPL clones to IFN-γ priming compared to non-RPL clones, the underlying mechanisms remain unclear. A comprehensive understanding of MSC-mediated immunomodulation^44^ and the pathophysiology of idiopathic miscarriages^45^ is essential to determine whether the genomic background of the fetus influences the immunomodulatory capacity of fetal MSCs. This underscores the importance of further exploring the genomic and epigenetic underpinnings of immunoregulatory mechanisms in RPL to better understand its pathophysiology and identify potential therapeutic targets.

Our study focused on the paracrine functions of AF-MSCs, particularly their role in shaping the immune microenvironment and influencing key mediators of T helper 1 (Th1) and T helper 2 (Th2) responses, such as Treg cells and macrophage subsets. We observed significant differences in Treg proportions between the RPL and non-RPL groups, particularly under the control condition and the 100 IU/mL-72 h IFN-γ treatment. Notably, MSC-CM derived from the RPL group induced fewer Treg cells compared to the non-RPL group (Fig. 5A). This suggests that the RPL-derived MSC secretome may have a reduced capacity for Treg induction, likely reflecting deficiencies or dysfunction in active mediators essential for immune tolerance in this group.

Interestingly, Treg generation was higher in the TGF-β1-supplemented basic medium (BM) compared to the MSC-CM control group. Given that TGF-β1 is a potent inducer of Treg differentiation from PBMCs^46^, this observation highlights the dominant role of TGF-β1 in driving Treg expansion independent of MSC-derived signals. A similar trend was evident in the Treg/CD4⁺ ratio (Fig. 5B), where the RPL group consistently showed lower ratios under both the control and IFN-γ-treated conditions. Since an elevated Treg/CD4⁺ ratio reflects enhanced immune tolerance and suppression of effector T cell proliferation, these findings may indicate an intrinsic immune dysregulation in RPL patients, as reported in reproductive and tumor immunology contexts^47–50^.

AF-MSC-derived secretomes, including extracellular vesicles (EVs), cytokines, and microRNAs, are well-documented for their immunomodulatory capabilities^51^. Recent evidence shows that MSC-derived EVs, particularly from IFN-γ-primed cells, can enhance FOXP3 expression in Tregs via microRNAs such as miR-139-5p and miR-214-5p^52^. The discrepancy in Treg induction between the RPL and non-RPL groups observed here could reflect differences in the bioactive composition of their respective MSC-CM.

Beyond Tregs, macrophage polarization was another key focus of our study. Circulating monocytes respond to microenvironmental cues by differentiating into pro-inflammatory M1 macrophages or anti-inflammatory M2 macrophages. M1 macrophages (CD68⁺CD80⁺) are typically involved in Th1-driven immune responses, while M2 macrophages (CD68⁺CD206⁺) promote Th2-mediated immune regulation and tissue repair^53,54^. MSCs are widely recognized for their capacity to modulate macrophage phenotypes, frequently inducing a shift toward the M2 phenotype through secreted immunoregulatory mediators such as TGF-β, IL-10, and PGE2^55,56^. Rather than isolating macrophages prior to analysis, we assessed polarization directly within the PBMC population. This approach allowed us to preserve the natural cellular interactions that occur between monocytes, T cells, and MSC-derived signals, thereby providing a more physiologically relevant model. Additionally, we employed CD68, a well-established marker for monocyte-derived macrophages in vitro^57^, to identify macrophages within the PBMC population.

In this study, we aimed to elucidate the paracrine effects of MSC-CM on macrophage polarization, particularly focusing on the immunomodulatory influence of both unstimulated and IFN-γ-preconditioned MSCs on PBMCs. To establish a consistent baseline for macrophage differentiation, all MSC-CM experimental groups were compared to cultures in basic medium (BM, RPMI 1640) supplemented with TGF-β1. TGF-β1 is known to enhance monocyte survival and plasticity, promoting the differentiation of non-activated macrophages into the M2 phenotype^46^. By using TGF-β1-supplemented medium as a control, we aimed to compare MSC-CM-induced effects on M2 polarization against a standardized M2-favoring environment.

Our results revealed a distinct pattern in macrophage polarization influenced by both the source of MSCs (RPL vs. non-RPL) and the treatment condition. Under control conditions, MSC-CM from non-RPL donors primarily induced an M1 phenotype, while RPL-derived MSC-CM favored M2 polarization (Fig. 5C and D). In contrast, following IFN-γ preconditioning (100 IU/mL-72 h), non-RPL MSC-CM promoted M2 polarization, while RPL MSC-CM shifted toward an M1 phenotype. These findings suggest that AF-MSCs respond to IFN-γ in a source-dependent manner, and that the immune background of the donor may shape the MSC secretome’s immunomodulatory capacity. Interestingly, the pattern of Treg induction partially mirrored the M2 macrophage trend: both were enhanced by non-RPL MSC-CM under IFN-γ preconditioning, suggesting the existence of shared or complementary regulatory pathways. However, the inverse relationship in the control condition (higher Treg levels in non-RPL but higher M2 macrophages in RPL) underlines the complexity of immune network interactions and the distinct pathways that govern Treg expansion and macrophage polarization^58^. We also observed time-dependent differences in the induction of a mixed M1/M2 phenotype (CD80⁺CD206⁺). This intermediate state, which reflects the inherent plasticity of macrophages in response to fluctuating environmental cues^54,59^, was more pronounced at 24 h in the non-RPL group, but shifted toward the RPL group at 72 h (Fig. 5E). This temporal variability further emphasizes the dynamic nature of MSC-mediated immune regulation.

Another key observation was that IFN-γ preconditioning did not consistently enhance M2 polarization, as might be expected from prior reports showing IFN-γ primes MSCs to produce anti-inflammatory mediators^60^. In our study, IFN-γ-treated MSC-CM led to an increased proportion of CD68⁺CD206⁺ M2 macrophages compared to control. Conversely, in the RPL group, M2 polarization decreased under IFN-γ treatment, except for the 20 IU/ml 72 h condition. This divergence suggests that the immunomodulatory effect of IFN-γ-primed MSCs may be altered in the context of RPL, potentially reflecting patient-specific immune backgrounds rather than MSC conditioning alone. It is also important to note that Tregs and M2 macrophages, while often functionally linked in maintaining immune tolerance, do not always correlate proportionally, as their induction is governed by partially overlapping but distinct regulatory pathways^50,58,61^. These findings underscore the importance of considering both MSC source and patient-specific factors when evaluating MSC-mediated immunomodulation. The unexpected results highlight the complexity of MSC-mediated immunoregulation, influenced not only by external stimuli like IFN-γ but also by the source of MSCs and the immune background of the patient samples.

This study has several limitations that should be acknowledged. The absence of additional control conditions — such as PBMC cultures in cytokine-free medium — limits our ability to fully distinguish the direct effects of MSC-CM from background immune plasticity. Moreover, while IFN-γ levels were standardized across all MSC-CM conditions, its well-established role in promoting M1 polarization and modulating Treg function^62,63^ cannot be entirely excluded as a confounding factor influencing the observed immune profiles. Additionally, although immune cell phenotypes were assessed using surface markers, secreted proteins and EVs cargo were not directly quantified, which limits the mechanistic depth of our conclusions. Functional validation assays, including T cell proliferation suppression tests, were also not performed, which would have clarified the biological relevance of the immunomodulatory shifts observed. Finally, the exploratory nature of the study and limited sample size necessitates cautious interpretation, and future research using larger cohorts, comprehensive molecular profiling, and broader experimental models will be essential to confirm and expand these findings.

Despite these limitations, our study addresses a novel and previously unexplored question. The findings underscore the need for further investigation into whether developmental defects observed in children born to couples with a history of RPL are reflected in AF-MSC characteristics and other cell types. Future research employing high-throughput techniques, such as RNA-seq and proteomics, along with rigorous functional assays, will be essential for validating and expanding these findings. By acknowledging these constraints, we aim to provide a transparent interpretation of our results and encourage further exploration of this promising research avenue.

## Conclusion

This study investigated the hypothesis that the immunomodulatory response of AF-MSCs to IFN-γ priming is influenced by their source, specifically comparing AF-MSC clones derived from healthy pregnancies and those with a history of idiopathic RPL. Our findings revealed significant differences between the two groups in their response to IFN-γ stimulation. AF-MSCs from the RPL group required higher doses of IFN-γ to induce immunomodulatory markers compared to the control condition. Conversely, AF-MSCs from the non-RPL group showed upregulation of these markers at lower doses but displayed decreased responsiveness or no significant changes at higher doses. In addition, the MSC-CM from the two groups exhibited distinct effects on immune cell modulation. Specifically, non-RPL-derived MSC-CM showed a greater capacity to induce Treg cells and anti-inflammatory M2 macrophages under specific treatment conditions, whereas RPL-derived MSC-CM demonstrated a stronger induction of pro-inflammatory M1 macrophages under comparable conditions. These observations underscore the differential immunomodulatory capacities of AF-MSC clones, highlighting the influence of their origin on the secretion of bioactive mediators and the subsequent modulation of immune responses. Our findings support the hypothesis that dysregulated immune pathways may play a critical role in the pathophysiology of idiopathic RPL. However, further studies are required to validate these results and elucidate the underlying mechanisms. High-throughput sequencing and functional analyses with larger sample sizes and more rigorous experimental designs will be essential to uncover the specific immune pathways and mediators involved. Such efforts could pave the way for developing targeted therapeutic strategies to address immune dysregulation in idiopathic RPL.

## Electronic supplementary material

Below is the link to the electronic supplementary material.

Supplementary Material 1

## Data Availability

All data will be available on request from the corresponding author.
