# Supplementary material for: Immunomodulatory effects of primed amniotic fluid-derived mesenchymal stem/stromal cells with IFN-γ from unexplained recurrent miscarriage sources
Source: Sci Rep. 2026 Jan 3;16:281. doi: 10.1038/s41598-025-01799-1 (PMC12769564; doi:10.1038/s41598-025-01799-1)
Supplement: Supplementary file 1 — Supplementary Material 1 [file 41598_2025_1799_MOESM1_ESM.docx]

**Immunomodulatory Effects of Primed Amniotic Fluid-Derived Mesenchymal Stem/Stromal Cells with IFN-γ from Unexplained Recurrent Miscarriage Sources**

**Supplementary Files**

**Supplementary** **Table 1)** The clinical characteristics of mothers and fetuses sampled for human amniotic fluid (hAF).

|  | non-RPL group | | | RPL group | | | |
| --- | --- | --- | --- | --- | --- | --- | --- |
| hAF samples | Maternal age (years) | Gestational age (weeks) | Fetal karyotype | Maternal age (years) | Gestational age (weeks) | Fetal karyotype | Number of miscarriages |
| S1 | 30 | 18 | 46, XY | 27 | 17 | 46, XX | 3 |
| S2 | 28 | 17 | 46, XX | 33 | 18 | 46, XX | 5 |
| S3 | 29 | 16 | 46, XY | 34 | 18 | 46, XY | 4 |
| S4 | 31 | 18 | 46, XY | 30 | 18 | 46, XY | 3 |
| S5 | 30 | 18 | 46, XX | 32 | 16 | 46, XY | 3 |


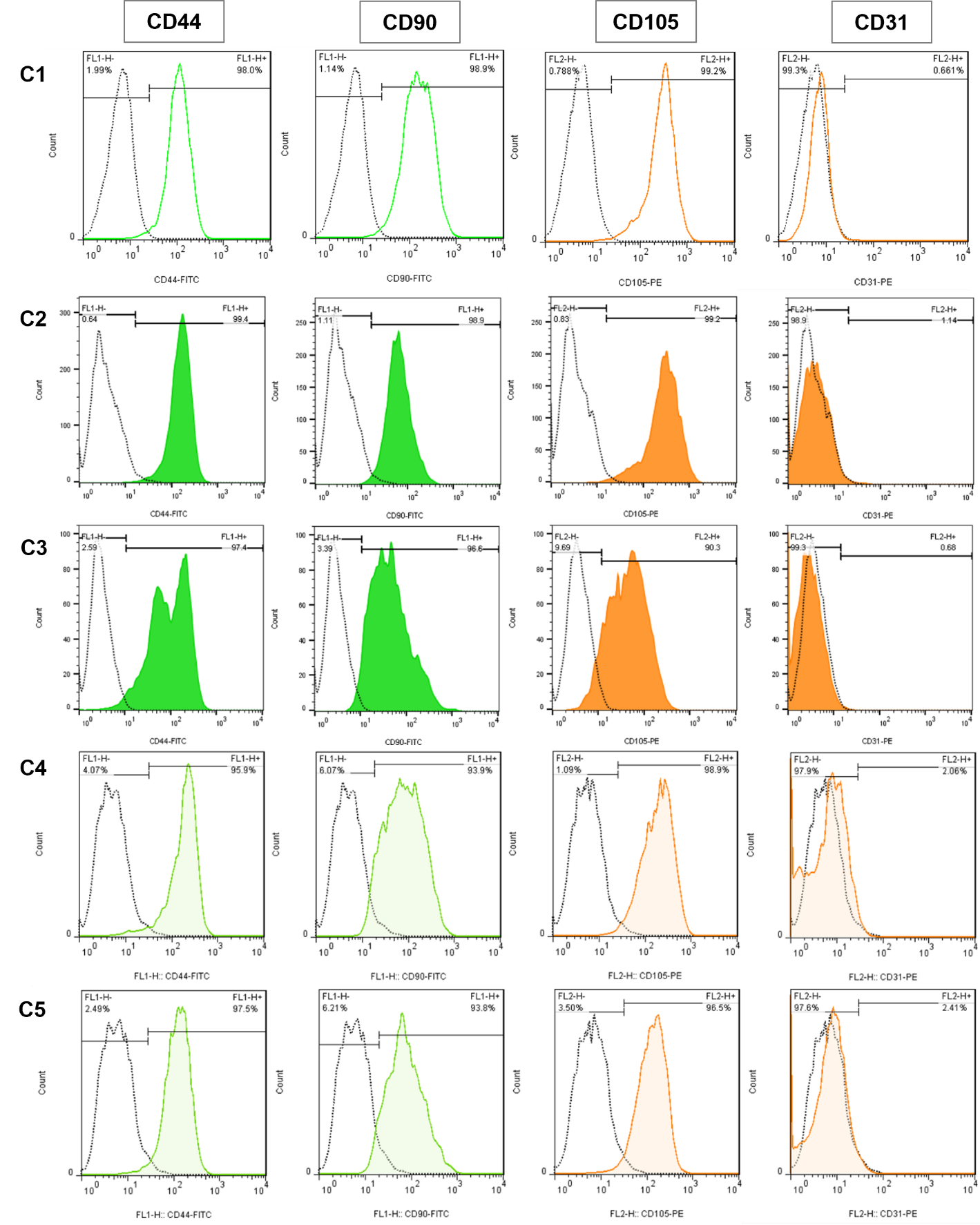


**Supplementary** **Figure 1)** Flow cytometry analysis of AF-MSCs from the non-RPL (healthy control) group at the 3^rd^ passage. As indicated above the histogram columns, they display the expression levels of CD44, CD90, and CD105 (positive MSC markers) and CD31 (negative marker) from left to right. Rows correspond to clones 1 to 5 (C1 to C5), presented from top to bottom.


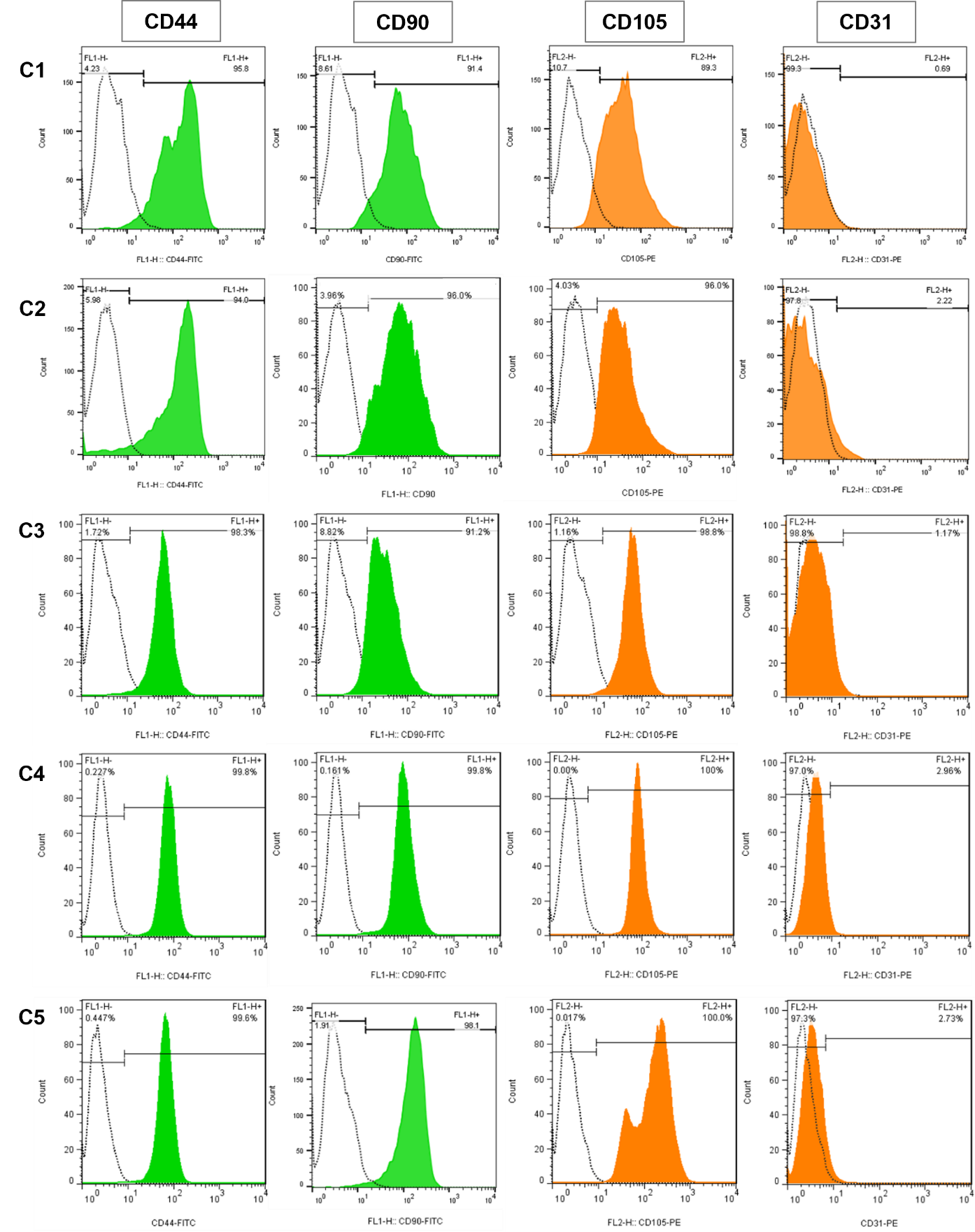


**Supplementary Figure 2)** Flow cytometry analysis of AF-MSCs from the RPL group at the 3^rd^ passage. As indicated above the histogram columns, they display the expression levels of CD44, CD90, and CD105 (positive MSC markers) and CD31 (negative marker) from left to right. Rows correspond to clones 1 to 5 (C1 to C5), presented from top to bottom.


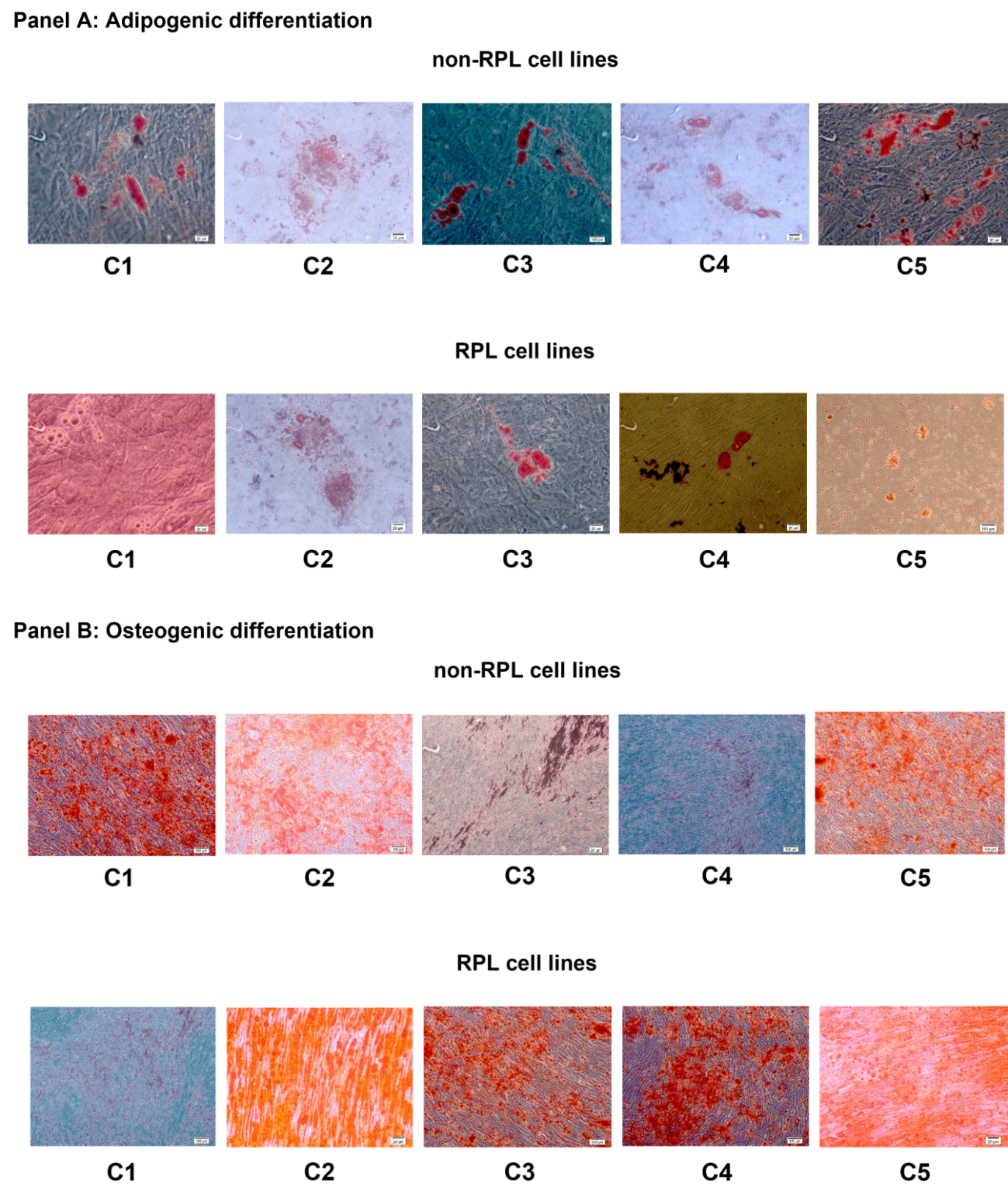


**Supplementary Figure 3)** Characterization of clones derived from amniotic fluid samples related to non-RPL (C1-C5) and RPL (C1-C5) groups regarding their mesodermal differentiation capacity at 3^rd^ passage following three weeks of culture under differentiation media. The results are depicted in two panels: (**A**) induced adipocytes in which lipid droplets are visible after oil red O staining; (**B**) induced osteocytes in which calcium deposits were confirmed by alizarin red S staining.


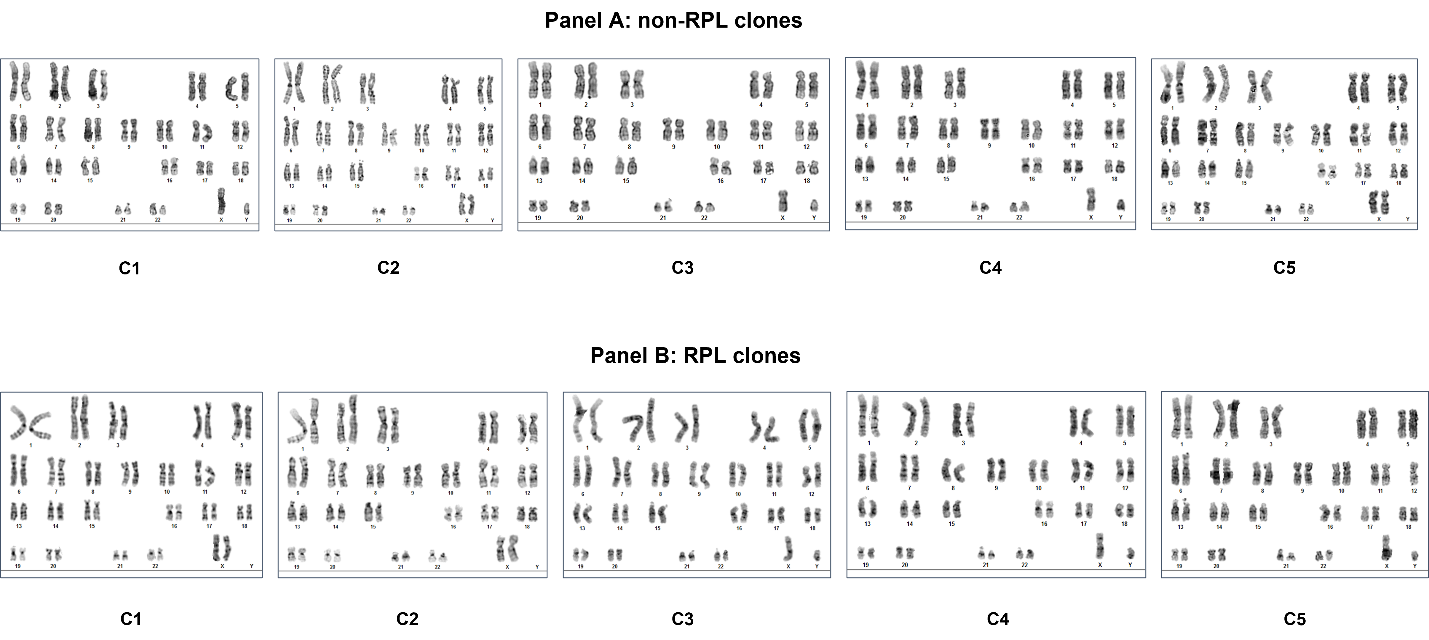


**Supplementary Figure 4)** Karyotype analysis of AF-MSCs at 3^rd^ passage; C1 to C5 represent the results of clones 1 to 5, respectively. The results are depicted in two panels: **Panel A**: non-RPL clones; **Panel B**: RPL clones
